# Impact of leading plant research centers of excellence on the scientific and socio-economic development in Europe

**DOI:** 10.3389/frma.2026.1770226

**Published:** 2026-04-16

**Authors:** Petar Kazakov, Dimo Atanasov, Rositsa Beluhova, Marina Mircheva-Topalova, Asya Ivanova, Vesela Kazashka, Tsanko Gechev

**Affiliations:** 1 Department of Molecular Stress Physiology, Center of Plant Systems Biology and Biotechnology, Plovdiv, Bulgaria; 2 Department of Economics, Agricultural University of Plovdiv, Plovdiv, Bulgaria; 3 Department of Molecular Biology, Plovdiv University, Plovdiv, Bulgaria

**Keywords:** centers of excellence, plant biology, plant biotechnology, plant metabolomics, systems biology

## Abstract

**Background:**

Centers of Excellence (CoEs) are key instruments for advancing scientific excellence, innovation, and socio-economic development in Europe.

**Methods:**

The paper compares three leading European Centers of Excellence in plant science: the Max Planck Institute of Molecular Plant Physiology (Potsdam-Golm, Germany), the Center for Plant Systems Biology at the Flanders Institute of Biotechnology (Gent, Belgium), and the Center of Plant Systems Biology and Biotechnology (Plovdiv, Bulgaria). The survey explores their organizational structures, funding sources, scientific focus and impact on the regional socio-economic environment.

**Results:**

All centers impose substantial influence on the scientific advancement of their countries. The study indicates that the contributions of these institutions extend beyond national borders, supporting scientific progress at the European and global level.

**Conclusion:**

In addition to research impact and innovation potential, these institutions enable socio-economic development of their regions by improving local infrastructure, creating jobs, promoting a favorable business environment, and facilitating stronger linkages between academia and industry.

## 1 Introduction

Europe needs constant research and innovation in order to stay the forefront of the World's scientific and technological development, as well as to maintain its attractiveness for international talent and the leading position in the global human social and economic development (Borlaug, 2016; Reichert and Furlong, 2014). The European Governments have recognized the need of Centers of Excellence (CoE) as drivers of this development and many European countries are investing heavily in development and sustainability of such centers, which are understood as institutional ecosystems that promote excellence in research, innovation, and education (Kitagawa, 2010). These centers not only generate new knowledge but also build capacity by fostering research-derived innovations (Hellström, 2011, 2012). Such CoE are aligned with scientific and technological progress, as well as with wider societal and policy objectives (Aksnes et al., 2012; Langfeldt et al., 2015). CoEs focus on high-potential and innovative fields in science and industry (Fischer et al., 2001; Luukkonen et al., 2006).

The organizational structure, teams, and collaboration within CoEs are essential (Schmidt et al., 2003). Studies suggest that flat organizational structures with low bureaucracy enhance performance (Hemlin et al., 2004; Heinze et al., 2009). Research autonomy is another critical factor influencing CoE effectiveness (Hollingsworth, 2002; Hollingsworth and Hollingsworth, 2000). Regarding funding, resource resilience and flexibility are important drivers of high-impact research (Hemlin et al., 2004; Laudel, 2006; Heinze et al., 2009). Funding also indirectly impacts performance, as seen in publication output, citations, and human capital development (Latour and Woolgar, 1986; Zuckerman, 1987). The OECD (2014) notes that CoEs with larger budgets tend to engage more in external cooperation, linking funding to interdisciplinary collaboration that fosters innovative research and long-term scientific impact. Positive correlations between collaboration and productivity have been documented (Katz and Martin, 1997; Narin et al., 1991; Orr et al., 2011).

The founding EU member states have invested heavily in such CoE, which correlated positively with their technological and socio-economic development. For example, the 84 institutes of the Max Planck Society, the 96 Leibnitz institutes, the 75 Fraunhofer research institutes, and the Helmholz Centers of Excellence in Germany are the backbone of the German research landscape. Collectively, their budgets total 18.81 Bln EUR for 2024 (Max Planck Society, 2024; Leibniz Association, 2024; Fraunhofer-Gesellschaft, 2024; https://www.helmholtz.de/en/). Likewise, the Flemish Government in Belgium has invested heavily in Centers of Excellence of the Flanders Institute of Biotechnology (VIB). The VIB budget for 2024 is 170 Mln EUR, 50% from which came from the Flemish Government, 15% from the Belgian Federal Government, 20% international grants, and the rest from the industry (https://vib.be/en/about/annual-reports/annual-report-2024).

The new EU member states, most of which in South-Eastern Europe, are lagging behind in both investment and R&I development. As a consequence, there is a significant brain migration from South-East to North-West, resulting in notable disparities between the two regions. To counteract this disparity in knowledge and technology development, the European Commission launched the new Teaming initiative for establishment of new CoEs in the lagging so called Widening countries, in partnership with advanced partners which are located mainly from North-West. The new CoE should maintain the same scientific excellence as their partners in the advanced countries with higher research and innovation index, but they will foster the local talents, accommodate returning fellows, and link the knowledge between the borders. Such an example is the newly founded center of Plant Systems Biology ad Biotechnology (CPSBB) in Plovdiv, Bulgaria, which was founded by scientists who returned from the Northern Europe with help of partners from the North (Germany). The development of CPSBB in South-Eastern Europe and its comparison with similar centers from North-Western Europe is presented here as a case study which demonstrates the positive example of knowledge circulation between the regions.

Here we review three leading European CoE in the field of Plant Biology: two of them in founding EU member states (Center for Plant Systems Biology at the Flanders Institute of Biotechnology in Gent, Belgium and the Max Planck Institute of Molecular Plant Physiology in Potsdam-Golm, Germany), both located in North-Western Europe, and CPSBB, located in Bulgaria, South-Eastern Europe. Our in-depth analysis highlights the significance of these centers for the science and technology of their respective host countries and demonstrates their importance for the socio-economic development of their regions.

## 2 Methodology

### 2.1 . Theoretical and methodological framework

This study uses a comparative case study methodology (Yin, 2013; George and Bennett, 2005) to systematically analyze three leading European CoEs in plant systems biology. These were selected based on their scientific impact, focus on molecular and systems approaches in plant biology, specialization, governance models, and participation in European frameworks.

The comparison follows analytical dimensions adapted from Bozeman and Boardman (2014), OECD (2019), and the Piwowar et al. (2018), enabling assessment of research performance, institutional embeddedness, and socio-economic value. The analysis combines detailed case descriptions with cross-case synthesis (Miles and Huberman, 1994). Results are presented in a comparative matrix to identify patterns and draw conclusions. Data was collected via document analysis and secondary sources, including institutional strategies, annual reports, bibliometric data (Scopus, Web of Science, institutional databases), project participation, and policy and funding reviews.

### 2.2 . Sources of information

The information sources employed in this research comprised databases such as Scopus (https://www.scopus.com/) and Web of Science (WoS, https://www.webofscience.com/), as they have extensive thematic coverage and recognition within the scientific community. Both databases are multidisciplinary, index scientific literature from peer-reviewed journals, conference proceedings, and patents, and encompass a wide range of subject areas. WoS has high-impact scientific publications and provides tools for analyzing citations and trends in academic production. The selection of these databases is based on their excellent reputation, consistent updating, and rigorous indexing of scientific publications (including high-impact journals). This study adopts a multi-dimensional approach to research impact assessment, taking into account the well-documented limitations of journal impact factors. As shown in the literature, impact factors are not representative of individual articles, correlate poorly with article-level citation performance, and are strongly influenced by field-specific publication and citation practices, editorial policies, and database coverage biases (Seglen, 1997). At the same time, despite these limitations, the journal impact factor remains a widely used and relatively standardized indicator of journal visibility. In this study, it is therefore used as one component of the assessment of publication quality. Impact factor is complemented by indicators related to open access dissemination models, peer review practices, and international collaboration patterns, reflecting evidence that research impact is strongly influenced by accessibility and dissemination pathways (Piwowar et al., 2018).

In addition, we used the CORDIS database, which belongs to the Research and Innovation platform of the European Commission and serves to provide comprehensive information about EU research and Development projects (https://cordis.europa.eu/).

Several national and international databases and web sites of national science funds and Ministries of Education and Sciences of EU member States were also used, such as the Bulgarian Ministry of Education and Science (MES, https://web.mon.bg/en/), Bulgarian National Science Fund (BNSF, https://bnsf.bg/), Flemish Research Foundation (FWO, https://www.fwo.be/en/), the Federal Ministry of Research, Technology, and Space of Germany (BMFTR, https://www.bmftr.bund.de/EN/), and the German Research Foundation (DFG, https://www.dfg.de/en). These national web sites provide publicly available information about the research policies of the respective countries, research organizations including research institutes and universities, and project databases of past and ongoing projects.

We have also utilized the web sites of the three studied CoEs, namely the Max Planck Institute of Molecular Plant Physiology (MPIMP, https://www.mpimp-golm.mpg.de/2168/en), the Center for Plant Systems Biology at the Flanders Institute of Biotechnology (PSB-VIB, https://www.psb.ugent.be/), and the Center of Plant Systems Biology and Biotechnology (CPSBB, https://cpsbb.eu/). These sites provide up-to-date information about the organizational structure, the personnel, the publications, the other research, and other activities (e.g. communication, dissemination, networking) of the three centers of excellence.

### 2.3 . Data processing and analysis

This subsection describes the data processing and analytical approach used to assess the scientific impact of MPIMP, PSB, and CPSBB at both national and international levels. The data related to the organizational structures and the scientific foci was retrieved and processed from the web sites of the centers. The projects of the three centers were retrieved from the CORDIS database and analyzed by identifying the collaborating organizations and the numbers of projects they have with the three centers.

Bibliometric data for MPIMP, PSB, and CPSBB were extracted from the Scopus database for the period 2020–2024 using affiliation-based searches and verified institutional name variants. The analysis included peer-reviewed articles and reviews only. Citation counts were extracted excluding self-citations. Scientific impact indicators comprised total publications, total citations, average citations per publication, and period-bounded h-index values.

International collaboration data were obtained using Scopus Documents by country or territory analytics. Domestic co-authorships were excluded. For each institute, partner countries were ranked by frequency of co-authored publications. Countries with the highest shares were reported individually, while remaining partner countries were aggregated into an “Other” category. To ensure comparability across institutes with different publication volumes, data were normalized internally and expressed as the relative share of international co-authorships (%).

**Figure 1:**
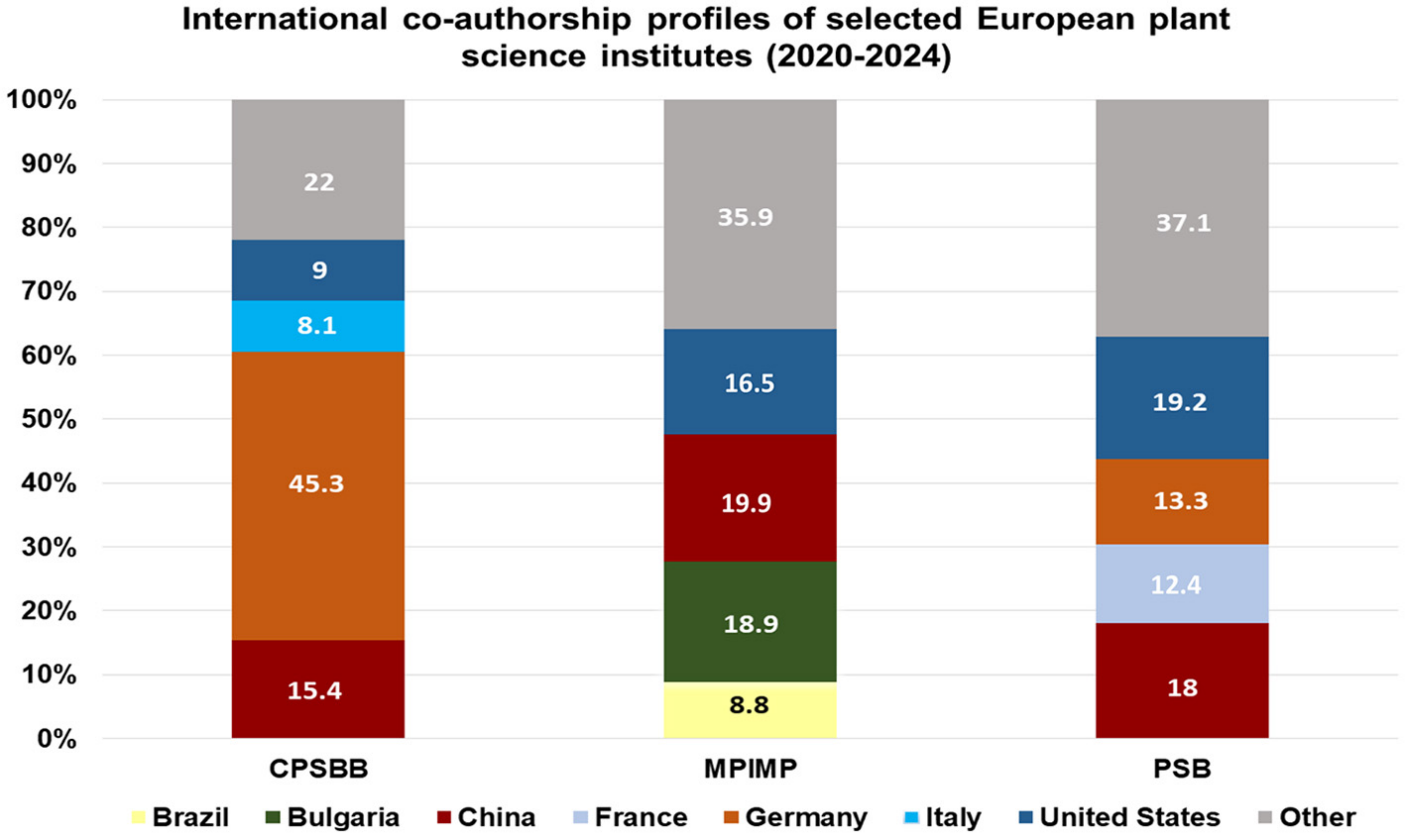
Distributions by countries of the research partner organizations of CPSBB, MPIMP and PSB. The data is from SCOPUS database (www.scopus.com).

The processed data were visualized using a 100% stacked bar chart to facilitate visualization (Figure 1).

Co-authorship networks were constructed as bipartite graphs linking research centers and countries, where edge weights represent the number of co-authored publications. Network analysis and visualization were performed using graph-based network analysis software, applying force-directed (spring) layout algorithms to display collaboration intensity and structural patterns.

## 3 Results and discussion

### 3.1 . Description of the three leading plant science centers of excellence

The Max Planck Institute of Molecular Plant Physiology (MPIMP) is part of the Max Planck Society (MPG), a German association comprising of 84 institutes across all fields of science (Max Planck Society, 2024, https://www.mpg.de/24877983/mpg-annual-report-2024.pdf). 82% of the nearly 3 billion EUR MPG income is Governmental funding, the rest is project funding (EU, DFG, other funding agencies) and own funding (Max Planck Society, 2024). As all MPG institutes, MPIMP is funded primarily by the Government, with half of its Governmental funding coming from the German Federal Government and the other half from the Government of Land Brandenburg. MPIMP was founded in Potsdam, Germany, in 1994. Initially, it had 16 employees located in a building at the premises of Potsdam University (https://www.mpimp-golm.mpg.de/5672/portrait). Gradually, MPIMP grew to about 360 people, from all over the world, which are located in a new large complex in Potsdam Science Park. MPIMP is together with two other MPG institutes: Max Planck Institute of Colloids and Interfaces and The Max Planck Institute for Gravitational Physics (Albert Einstein Institute).

The mission of MPIMP is to understand the molecular and genetic mechanisms that control plant development, physiology and interactions with the environment. MPIMP investigates plants at various levels with a particular focus on three scales: interactions within plant cells, interactions of plant cells with each other, and interactions between plants and other organisms (pathogens, symbiotic fungi, animals, and plant-plant interactions). The vision of MPIMP has always been to be at the forefront of EU plant science research. MPIMP has three research departments: “Organelle Biology, Biotechnology and Molecular Ecophysiology”, “Plant Reproductive Biology and Epigenetics”, and “Root Biology and Symbiosis”, with several research groups in each of the tree departments. Foci of research include, but not limited to, physiology and genetics of cell organelles, molecular mechanisms of the evolution of plant genomes, seed formation of flowering plants, epigenetic regulation of plant reproduction, symbiosis between plants and mycorrhiza fungi, and plant metabolism. Since its establishment, MPIMP positioned itself at the forefront of the German plant science. MPIMP is performing strong fundamental science and is often ranked among the top institutes according in Nature Index or the Index of Highly Cited Researchers. According to the Clarivate Group, four MPIMP researchers are highly cited in 2025 (https://clarivate.com/highly-cited-researchers/).

The Center for Plant Systems Biology (PSB, https://www.psb.ugent.be/) in Gent, Belgium, is part of the Flemish Institute of Biotechnology (VIB), a research institute founded by the Flemish Government in 1995. In contrast with the Max Planck Institutes in Germany, which have their own research premises, the institutes of VIB operate in the campuses of six Flemish establishments: Gent University, University of Leuven, Interuniversity Microelectronics Center Leuven, University of Antwerp, Free University of Brussels, and Hasselt University (https://vib.be/en#/). Many of the VIB group Leaders, including the Group Leaders of PSB, are also Professors at the respective host universities. PSB is located in Tech Lane Ghent Science Park, Gent, Belgium, a science park operated by the University of Gent. Hence, PSB can be regarded also as a part of Gent University. Currently, PSB is employing more than 350 scientists, working on all aspects of plant biology.

The mission of PSB is to lead the Flemish and Belgium plant research in the fields of Plant Biotechnology, Plant Development, Stress Physiology, and Systems Biology and to translate the scientific excellence into benefits for society. PSB is a successor of the Laboratory of General Genetics, pioneered by Jeff Schell and Marc Van Montagu, known for its breakthrough research on the *Agrobacterium tumefaciens* mediated plant transformation and plant genetic engineering. The laboratory was integrated into the newly created Flanders Institute for Biotechnology (VIB) in 1995 and renamed Department of Plant Genetics, and later became PSB. The research at PSB is organized in 20 Research Groups: Advanced Cell Imaging, Bio-energy and Bio-aromatics, Bioinformatics and Evolutionary Genomics, Brassinosteroids, Cell Cycle, Computational Regulomics, Ecological Genomics, Evolutionary Systems Biology, Functional Interactomics, Functional Phosphoproteomics, Innovative Breeding, Inter-organelle Stress Signaling, Oxidative Stress Signaling, Plant Genome Editing, Programmed Cell Death, Rhizosphere, Root Development, Specialized Metabolism, Plant Growth Dynamics, and Vascular Development (https://www.psb.ugent.be/research). Like MPIMP, PSB is highly international and ranked high in the prestigious Science Indexes. According to the Clarivate Group, three PSB researchers are highly cited in 2025 (https://clarivate.com/highly-cited-researchers/).

The Center of Plant Systems Biology and Biotechnology (CPSBB) in Plovdiv, Bulgaria, was founded as an independent research institute in 2015 (Gechev et al., 2024). In contrast with MPIMP and PSB, it is fully autonomous, not under the umbrella of any larger organization (not part of the Bulgarian Academy of Sciences). It was established with 15 Mil. EUR seed money from the Teaming initiative of the EU (project PlantaSYST), which aimed at resolving the science and technology disparities between the EU member states with advanced research and the EU member states with low research and innovation index, and additional 15 Mil. EUR co-funding from the Bulgarian Government (Kazakov et al., 2023). Initially with just six founding people, CPSBB has grown to 60 employees in 2025 (www.cpsbb.eu).

The mission of CPSBB is to become a leading EU plant science organization in the fields of plant biotechnology and systems biology, which links the Bulgarian universities and research institutes through collaborative research and services. The research performed by CPSBB focuses on plant development, stress physiology, biotechnology, and vegetable breeding, whereas the services provided by CPSBB include plant metabolomics and plant breeding. The vision of CPSBB is to act as a bridge between the academic institutions and the industry and to consolidate the plant research in Bulgaria for the benefits of society. The research at CPSBB is organized in seven Departments: Bioinformatics and Mathematical Modeling, Crop Quantitative Genetics, Molecular Stress Physiology, Plant Cell Biotechnology, Plant Development, Plant Metabolomics, and Vegetable Breeding (Gechev et al., 2020), covering virtually all major fields of plant biology. Similarly to MPIMP and PSB, CPSBB is highly international. According to the Clarivate Group, one CPSBB researcher is highly cited in 2025 (https://clarivate.com/highly-cited-researchers/).

### 3.2 . Scientific impact of MPIMP, PSB, and CPSBB at national and international levels

The three European centers of Excellence have profound impact on the science in their countries. This impact is measured by objective criteria such as peer reviewed publications in high and medium impact factor journals, independent citations of these publications, research projects, but also collaboration with universities, research institutes, and companies, as well as patents, utility models, and spin-off companies. This section presents a comparative assessment of the scientific impact of the three CoE with the other major non-university plant science research institutes in Germany, Belgium, and Bulgaria, based on Scopus bibliometric data for the period 2020–2024. The scientific performance is evaluated using total publications, citations excluding self-citations, average citations per publication, and the period-bounded h-index. In addition, international co-authorship patterns are used to contextualize the global integration of the analyzed institutes. For each country, the selected institute is positioned within its national research landscape through comparison with other major plant science organizations active during the same period.

MPIMP produced 1,136 publications between 2020 and 2024 (average 227 per year) and accumulated 24,018 citations excluding self-citations, corresponding to 21.1 citations per publication and a period-bounded h-index of 67 (Table 1; www.scopus.com). Within the German context, MPIMP demonstrates scientific performance comparable to other major plant science institutes, including the Max Planck Institute for Plant Breeding Research (1,048 publications; h-index 69) and the Leibniz Institute of Plant Genetics and Crop Plant Research (1,023 publications; h-index 63). Although the Julius Kühn Institute exhibits a higher publication volume (1,544 publications), its citation efficiency is lower, reflecting differences in institutional research profiles. MPIMP accounts for 14.6% of the publication output of the eight German plant science institutes included in the analysis. International collaboration represents a substantial component of MPI-MP's research activity, with international co-authorships most frequently involving partners from China, the United States, and the United Kingdom (Figure 1).

**Table 1:** SCOPUS bibliometric data of MPIMP, PSB, and CPSBB for the period 2020–2024.

Center	Created	Funded by	Ecosystem embedded	Personnel	Total articles	Articles per year	Total citations	Citations per year	*h*-index
MPIMP	1994	Mainly by government	Potsdam science park	360	1,136	227	24,018	21.1	67
PSB	1995	Mainly by government	Gent science park	360	803	160.6	18,142	22.6	58
CPSBB	2015	Projects, government	Bioeconomy hub plovdiv	60	322	64.4	11,348	35.2	45

The topics of these scientific articles reflect the research departments and the research groups of MPIMP, and are related to all major fields of molecular plant physiology and plant development, including: Algal biology, Bioinformatics, Epigenetic Mechanisms of Plant Reproduction, Intercellular Macromolecular Transport, Mycorrhiza and Root Biology, Organelle Biology and Biotechnology, Photosynthesis, Plant Cell Biology and Morphodynamics, Plant Cultivation and Transformation, Plant Germline Antiviral Immunity, Plant Metabolomics, Receptor structures at the plant-microbe interface, Seed Development and Apomixis, Synthetic Biology, Systems biology and Mathematical Modeling, Translational Regulation in Plants, and Viral Replication and Plant Tolerance (https://www.mpimp-golm.mpg.de/2777973/research-groups). MPIMP cooperates with all major plant science centers in Europe, including PSB and CPSBB. MPIMP exhibits the largest, most diverse, and most globally distributed co-authorship network of the three centers, characterized by very high collaboration intensity with major scientific powers such as the United States, China, the United Kingdom, France, and Japan (Supplementary Figure 1). The network is highly centralized around Germany but maintains strong and balanced links across Europe, North America, Asia, and Oceania, indicating a leading role in international research collaboration and knowledge exchange. The broad spread of partnerships, including many lower-intensity ties, suggests that MPIMP functions as a key global hub that integrates multiple regional research systems rather than being dependent on a small number of strategic partners.

MPIMP's strongest collaborations are within its core scientific domains: Biochemistry, Genetics and Molecular Biology, and Agricultural and Biological Sciences. These areas account for most of the institute's publications. MPIMP also maintains strong, ongoing partnerships with the CPSBB and the University of Potsdam, fostering a stable regional collaboration in plant molecular biology, genetics, and systems biology. Outside of its core domains, MPIMP maintains high-intensity international collaborations with a select group of long-standing partners in Europe, China, and South America, including INRAE, the Chinese Academy of Sciences, Huazhong Agricultural University, the National Key Laboratory of Crop Genetic Improvement, and the Universidade Federal de Viçosa. These partnerships mainly focus on research themes in crop genetics, phenotyping, and plant biotechnology. Medium- and lower-intensity collaborations extend into more specialized or interdisciplinary areas, such as chemistry, computational biology, environmental sciences, and applied life sciences.

Scopus publication data shows that 900 out of 1,136 publications (79.2%) are classified as open access. Open dissemination is achieved through multiple routes, with Green open access the most prevalent (744 publications), followed by Gold open access (384 publications), Hybrid Gold (356 publications), and Bronze open access (117 publications). As for the other centers, these values provide an approximate but informative picture of open dissemination practices, while acknowledging overlaps between open access categories.

Another trend in recent years is open peer review, in which the identities of both reviewers and authors are disclosed and greater participation in the peer review process is enabled (Ross-Hellauer, 2017; Tennant et al., 2017). However, the percentage of open peer review is still marginal, not only for MPIMP, but for the vast majority of research institutes as well, hence its role in the peer review and publishing process is minimal.

MPIMP engages in a range of activities that extend its impact into broader academic and public spheres. MPIMP hosts weekly international seminars with guest speakers from research institutions worldwide, contributing to knowledge exchange and scientific context across disciplines. The institute also participates in community-oriented events, such as open days and guided tours, to make plant science accessible to students, school groups, and the interested public, including hands-on activities that introduce young people to scientific research.

PSB generated 803 publications during 2020–2024 (average 160.6 per year) and received 18,142 citations excluding self-citations, resulting in an average of 22.6 citations per publication and a period-bounded h-index of 58 (Table 1; www.scopus.com). Compared with other Belgian plant science organizations, PSB shows strong citation performance relative to its publication volume. ILVO produced 998 publications (h-index 56), while the University of Liège–Gembloux Agro-Bio Tech generated 714 publications (h-index 40). PSB accounts for 29.2% of the total publication output of the four Belgian plant science institutes analyzed. According to Scopus co-authorship data the international collaboration at PSB is concentrated in Agricultural and Biological Sciences (542 publications) and Biochemistry, Genetics and Molecular Biology (492 publications), which together form the core disciplinary foundation of the institute's scientific output. These related fields support a dense and highly internationalized collaboration network, defined by a strong institutional core. The most intensive co-authorship links occur with Ghent University, reflecting the integrated VIB–UGent model, as well as with a select group of major European and international research organizations, each contributing several dozen to several hundred joint publications. International co-authorship at VIB is characterized by strong collaborative links with research organizations in the United States, China, Germany, and South Africa (Figure 1). Beyond this core, PSB sustains a broad layer of medium-intensity collaborations, typically involving partners with approximately 20 to 40 joint publications. These collaborations include leading research institutions across Europe, China, and North America and are often associated with thematically focused research in plant molecular biology, genomics, metabolomics, and biotechnology, as well as interdisciplinary work at the interfaces of chemistry, computational sciences, and environmental research. The network analysis (VIB-PSB network is strongly internationally oriented but more selectively concentrated than MPIMP, with Belgium, the United States, China, France, Germany, and South Africa forming a clearly defined core of high-intensity collaborators (Supplementary Figure 1). This pattern indicates a collaboration strategy focused on deep, high-impact partnerships with key scientific countries and institutions, positioning VIB-PSB as a high-quality but somewhat more specialized international research hub rather than a broadly distributed global network.

Scopus data for PSB reveal that 736 out of 799 publications (92.1%) are classified as open access. Open dissemination is predominantly achieved through Green open access (712 publications), complemented by Gold open access (252 publications), Hybrid Gold (142 publications), and Bronze open access (229 publications). These values provide an approximate but informative picture of open dissemination practices, while acknowledging that open access classifications in Scopus are based on automated metadata and may involve overlaps between categories.

PSB complements its scientific publications with training and knowledge exchange activities, including workshops, courses, and international conferences that connect researchers with industry and policy stakeholders. In addition, PSB supports outreach initiatives such as public blogs, podcasts, and educational content aimed at making science more accessible, and it collaborates internationally through outreach entities like the UGent-VIB International Plant Biotechnology Outreach (IPBO), which fosters capacity building and scientific cooperation for sustainable agriculture in less developed economies.

CPSBB produced 322 publications during the analyzed period (average 64.4 per year) and received 11,348 citations excluding self-citations, yielding an average of 35.2 citations per publication and a period-bounded h-index of 45 (Table 1; www.scopus.com). Within the Bulgarian context, CPSBB substantially outperforms other plant science institutes, such as the Maritsa Vegetable Crops Research Institute (110 publications; h-index 10) and the Dobrudzha Agricultural Institute (29 publications; h-index 3). CPSBB contributes 28.7% of the total publication output of the seven Bulgarian institutes assessed. The CPSBB co-authorship network shows a more regionally and moderately diversified international profile, with a very strong core partnership axis centered on Bulgaria and Germany, followed by China and a smaller but consistent set of EU and neighboring partners (Supplementary Figure 1).

CPSBB's Scopus data indicate that 257 out of 322 publications (79.8%) are classified as open access. Within this group, open dissemination is achieved through multiple routes, including Green open access (215 publications), Gold open access (133 publications), Hybrid Gold (89 publications), and Bronze open access (23 publications). These values provide an approximate but informative picture of open dissemination practices, while acknowledging that open access classifications in Scopus are based on automated metadata and may involve overlaps between categories.

Beyond publications, CPSBB engages in training and knowledge exchange activities to strengthen human potential and societal impact. The Center collaborates with local universities and higher schools by hosting student visits and practical training activities, supports early-career researchers through workshops and seminars, and participates in international initiatives promoting researcher mobility. These activities complement publication-based impact by facilitating the dissemination and practical use of research knowledge beyond the academic community.

Scopus co-authorship data for CPSBB show that international collaboration is predominantly concentrated in Biochemistry, Genetics and Molecular Biology, which accounts for the center's primary scientific category (216 publications). Agricultural and Biological Sciences follow closely (188 publications). Together, these two related fields account for most of CPSBB's scientific output and shape the structure of its international collaboration network. For CPSBB, international collaboration is characterized by a concentration of international co-authorships, with Germany accounting for the largest share, followed by China and the United States (Figure 1). A small core group of partners is responsible for a significant share of joint publications, reflecting strong, recurring collaborative relationships, particularly with institutions in Germany (such as MPI and the University of Potsdam) and Bulgaria (the Bulgarian Academy of Sciences and the University of Plovdiv). Beyond this core group, CPSBB maintains a set of medium-intensity collaborative relationships, typically involving 7 to 15 joint publications per partner, primarily with institutions in China (Chinese Academy of Agricultural Sciences, the Chinese Academy of Sciences) and Israel (Ben-Gurion University of the Negev). These partnerships demonstrate sustained, thematically focused collaboration in specific domains of plant biology and crop improvement.

Additionally, CPSBB's collaboration network comprises numerous weak ties, typically involving 2 to 5 joint publications, and extends across Europe, Asia, North America, and Africa. Collaborations with African partners, in particular, are often part of time-limited international projects and capacity-building initiatives, which accounts for their lower publication intensity. While less frequent individually, these partnerships expand the centre's geographic reach and facilitate knowledge exchange and international cooperation beyond the core network.

Overall, MPIMP, PSB, and CPSBB are among the most productive plant science institutes in their countries, generating the largest percentage of scientific publications compared with the other plant science institutes. Furthermore, most of these articles are in medium and high impact factor reputed journals, and the citations of these articles are very high (from 22.6 to 35.2 citations per publication, Table 1).

International collaboration across the three centers exhibits a consistent structure. A small group of long-term partners, primarily in plant and life sciences, accounts for a substantial share of joint publications. In contrast, a broader set of partners contributes less frequently. Strong collaborative relationships are typically found in core research areas, including molecular plant biology, genetics, and biotechnology. Weaker connections are formed around specific projects, interdisciplinary initiatives, or capacity-building activities, thereby broadening both the thematic scope and geographical reach of the centers.

Following the recent policy of the European Commission‘s Framework Programmes (Horizon 2020, Horizon Europe) and striving for visibility, the majority of the publications are published as Open Access (from 79.2% for MPIMP to 92.1% for PSB). Taken together, these patterns suggest that open access practices across the centers are primarily shaped by shared European open science policy frameworks, funding requirements, and disciplinary publishing norms in plant science and biotechnology. The European Commission itself dedicates substantial resources for OA not only from the Horizon Europe's funded research projects, but also through initiatives such as the Open Research Europe publishing venue (https://open-research-europe.ec.europa.eu/). The limited visibility of Diamond (Platinum) Open Access publications in the outputs of the three research centers should be interpreted in light of both disciplinary publishing practices and structural features of academic indexing systems. As discussed by Fuchs and Sandoval (2013), Diamond Open Access—defined as non-profit, non-commercial publishing without fees for authors or readers—remains systematically underrepresented in major commercial citation databases, particularly in the natural and life sciences. In fields such as plant biotechnology, where leading journals are predominantly published by large commercial publishers and rely on APC-based Gold or Hybrid models, opportunities for Diamond Open Access publishing are structurally constrained. Consequently, the marginal presence of Diamond Open Access in the analyzed publication portfolios reflects broader characteristics of the scholarly publishing ecosystem rather than explicit institutional preferences or governance choices. This observation is consistent with recent European research policy discussions, which recognize the societal value of Diamond Open Access while also acknowledging the current structural and disciplinary barriers to its wider adoption in experimental and high-technology research fields.

Besides the above measurable parameters, the scientific impact should also include the numerous research collaborations with universities and other research institutes. All three CoEs have vast networks of collaborators all over the world and this research cooperation further maximizes the scientific impact of the three institutes. Figure 1 presents the countries distribution of the research partner organizations across the world, as documented by their joint publications (www.scopus.com).

In addition to the partnerships with universities and research organizations across the world (Figure 1), MPIMP, PSB, and CPSBB have established vibrant links with industry partners in the form of joint research projects, joint ventures, or economic activities (Table 2; Supplementary Table 1).

**Table 2:** Number of universities, research institutes, and companies which are partners of MPIMP, PSB, and CPSBB.

Centers of excellence	Number of partner universities, research institutes, and companies		
	Universities	Research institutes	Companies
MPIMP	28	36	30
PSB	90	101	62
CPSBB	30	37	24

The data is retrieved from the EU project database CORDIS (https://cordis.europa.eu/). Partner institutions for all CoEs are listed in Supplementary Table 1.

**Figure 2:**
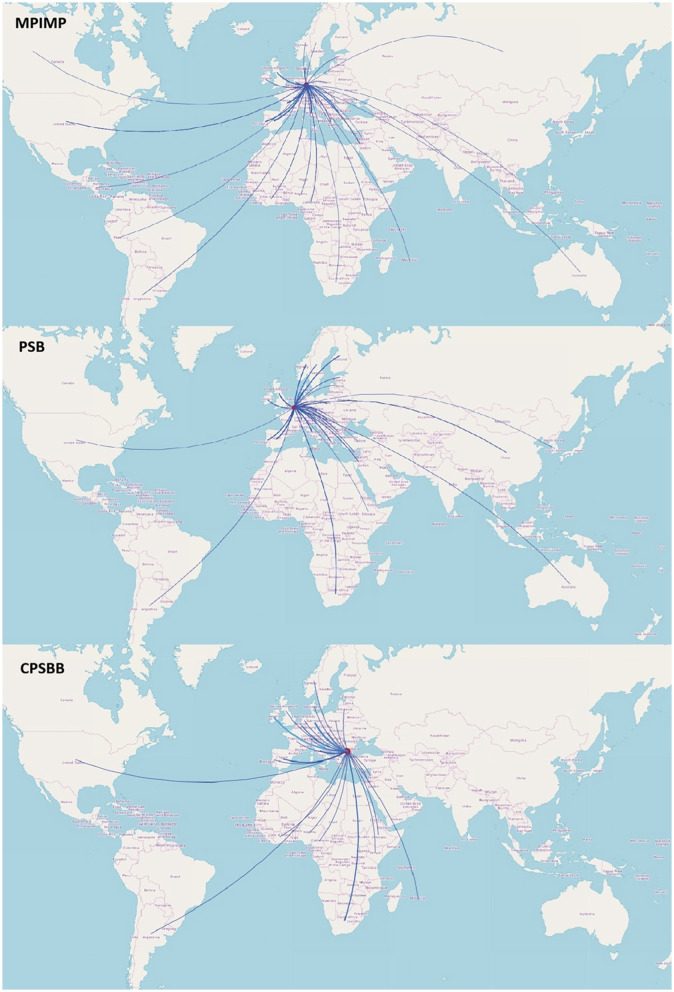
Geographical distribution of MPIMP, PSB, and CPSBB project partners by countries. The data is retrieved from the EU project database CORDIS, https://cordis.europa.eu/ (Map data available from OpenStreetMap, https://www.openstreetmap.org/copyright, under the Open Data Commons Open Database License [ODbL]).

The objectives and thematic foci of the projects implemented by the three centers mirror their research foci indicated in the scientific publications. Major scientific areas investigated in the projects include molecular physiology, plant development, plant metabolomics, synthetic biology, systems biology, bioinformatics, and vegetable breeding, to name a few. The geographical distribution of the project partners of the three centers is presented in Figure 2.

Most of the MPIMP projects are with partners in Europe, the top partner being the Spanish National Research Council (CSIC), INRA France, ENEA Italy, and the John Innes Center in United Kingdom (Supplementary Table 1; Figure 2). The top PSB project partners are also from Europe, including the Spanish National Research Council (CSIC), the Barcelona Supercomputing Center in Spain, Center National de la Recherche Scientifique (CNRS) France, Consiglio Nazionale delle Ricerche Italy, and the Masaryk University, Czechia. The top project partners of CPSBB are MPIMP, BioAtlantis Ireland, Sofia University, and the University of Potsdam, Germany (Supplementary Table 1). The networks established by the three institutes resembles the previous network analysis of agroecology projects in Europe, identifying France, Germany, Italy, Netherlands, Portugal, Spain, and United Kingdom as the most active countries in terms of project participation in the agri-food area (Iocola et al., 2023).

Together, these links positioned the three centers in the core of plant biotechnology hubs in Germany, Belgium, and Bulgaria. In particular, MPIMP is intertwined with the University of Potsdam and is essential part of Science Park Golm; PSB is part of the Gent University and Gent Science Park, and CPSBB has deep collaboration with research establishments in Plovdiv, such as the University of Plovdiv, the Agricultural University of Plovdiv, Maritsa Vegetable Crops Research Institute, and the Institute of Microbiology.

### 3.3 . Impact on the regional socio-economic development of the European countries

The three CoEs are large employees in their cities, directly creating jobs for the biotechnology industries in the regions. More importantly, however, they stimulate other businesses in their respective regions by cooperating with the local companies, institutes, and universities (Table 2), thus contributing even more to the economic developments of their regions. MPIMP and PSB are at the core of the Science Park Potsdam (https://potsdam-sciencepark.de/en/) and the Gent Science Park (https://www.techlane.be/), respectively. The two science parks accommodate hundreds of biotechnology companies, other research institutes, and the local universities. They have transformed the regions of Potsdam and Gent into world-class science and technology environments which further boost the economy of the regions.

The establishment of Potsdam University in 1991 and the MPIMP 4 years later had a deep impact on the socio-economic development of Potsdam-Golm. Golm was a small village with limited infrastructure and no economy. After the decision of Land Brandenburg, together with the German Federal Government, create the University of Potsdam and to invest in the region, the infrastructure was gradually developed (roads, housing complexes, shops), and several institutes and companies populated the growing Potsdam Science Park—to become what is now one of the largest concentrations of research institutes and talents in Germany. Nowadays, Science Park Potsdam is home of three Max Planck institutes (including MPIMP), two Fraunhofer institutes, the largest campus of the University of Potsdam, the Brandenburg Main State Archive, and 36 companies (https://potsdam-sciencepark.de/en/articles/companies/), 18 of which in the field of (plant) biotechnology and medicine: ArtemiFlow GmbH, Biotx.ai GmbH, CanChip GmbH, Evidentic, GlycoUniverse GmbH & Co KGaA, Hybrotec GmbH, MetaSysX GmbH, Mimi-Q, Molzym GmbH & Co. KG, Nanolytics GmbH, new/era/mabs, Pharmilabs, POROUS GmbH, QMEDIS Analytics GmbH, Quartett Immunodiagnostika, Biotechnologie + Kosmetik Vertriebs GmbH, Remi Health GmbH und MOMA Testlabor GmbH, Ripac-Labor GmbH, and Signature Diagnostics GmbH (Supplementary Table 1). Some of these companies are spin-offs of MPIMP. Altogether, the research establishments and the companies in Potsdam Science Park are the largest employers in the region. Hundreds of jobs are offered in the fields of Biochemistry, Bioinformatics, Biomedical Science, Biotechnology, Genetics, Metabolomics, Molecular Biology, and Systems Biology. While significant percentage of the jobs are for researchers, such as postdoctoral scientists and PhD students, there are also many jobs for technicians, IT specialists, and support personnel. In MPIMP and the two other MPG institutes, there are more than 200 people technical and support personnel, with many more at the University of Potsdam and the other institutes and companies in Potsdam Science Park.

In Gent, similar development took place about 30 years ago, when Gent University together with City of Gent and the Flemish Government organized the Gent Science Park, with the Center for Plant Systems Biology (PSB) in its core. Currently, Gent Science Park accommodates 11 university labs, 12 public research centers, 8 industrial pilots & test facilities and more than 90 knowledge intensive start-ups, academic & corporate R&D centers (https://www.techlane.be/about/). There are six Innovation Clusters in the park, including the Plant Biotech cluster focused on research areas such as Plant growth and crop yield, Drought tolerance, Biostimulants, CRISPR/Cas genome editing, and Bioinformatics. Companies in the field of (plant) biotechnology and biomedical research, some of which spin-offs of Gent University or/and PSB, are Amalus Therapeutics, Animab, Apcor R&M, Aphea.Bio, Barry Callebaut, BASF, BIO INX, Confo Therapeutics, Exevir, Eurofins Amatsigroup, Fujirebio, Gulliver Biomed, Harpago, Janssen Farmaceutica, Landis+Gyr, Legend Biotech, Life-ID, Ligentec, MRM Health, Nano Gold, Obelisc Bio-accelerator, OCAS, OneTouch Technology, Orionis Biosciences, Thermo Fisher, PKS, Primoris, ProDigest, Protealis, QustomDot, Riedel Communications, Sanofi, Sensnet, Sentea, Sirris, Sofico, Solvus, Syngenta. Hundreds of jobs for researchers (postdoctoral scientists, group leaders, PhD students), technical, and support personnel are offered by the research departments of PSB and even more by the companies located in the Gent Science Park. The jobs are in the areas of Agroindustry, Bioinformatics, Biomedical and Pharmaceutical Research, Biological Sciences etc. Importantly, the number of jobs for the technicians and support personnel are also in the range of several hundreds, making the Gent Science Park together with the University of Gent the largest employer in the region.

CPSBB, a much younger research institute, develops itself like the other two older partner institutes in Germany and Belgium, and is forming a local biotechnological hub in Plovdiv, Bulgaria. CPSBB, for example, has contracts, joint projects, or cooperation agreements with local companies (AgroHub, Bevine, GeosemSelect, Ondo, Opora Zaden, Seed House Sadovo, TechnoLogica), research institutes (Fruit Growing Institute, Institute of Microbiology, Institute of Molecular Biology and Biotechnology, Maritsa Vegetable Crops Research Institute), and universities (Agricultural University of Plovdiv, Medical University of Plovdiv, Plovdiv University, University of Food Technology), thus creating a local multi-billion biotechnology cluster (Ruseva et al., 2025; Supplementary Table 1). The jobs provided by CPSBB are for researchers in the fields of Agronomy, Bioinformatics, Biology, Biotechnology, Genetics etc. but also IT experts as well as technical and support personnel. Both senior and junior researchers are being employed by CPSBB. In 2024–2025, CPSBB was one of the largest employers for students graduating the University of Plovdiv. Since 2023, CPSBB is also part of the Plovdiv Regional Bioeconomy Hub, together with the Agricultural University of Plovdiv, University of Food Technology, and several local companies. More importantly, CPSBB has a vast international network of more than 100 partners, which includes renown universities, research institutes, and global companies from the plant biotechnology and medical biology sectors of the economy.

## 4 Conclusion

The three centers have established themselves as plant science leaders in their respective countries, contributing the highest percentage of research articles compared with the other plant institutes. The missions and the visions of the three centers have always been to be at the forefront of the EU plant science and the impact they have made fully aligns with their mission. Their notable scientific impact is further substantiated by their vast international network, including renown universities, research institutes and companies worldwide. Their impact on the economy is measured by creating thousands of jobs, directly or indirectly through stimulating local businesses and catalyzing the formation of regional science parks in Potsdam, Gent, and Plovdiv. The local science parks and the links with the local universities create a vibrant ecosystem of youth and technology which attract massive investment in the regions and drive the socio-economic development. The regions of Potsdam-Golm, Gent, and Plovdiv have now the reputations as global science and technology centers attractive to international talents and multi-national companies.

This study clearly demonstrates the positive impact of the three centers on the scientific and socio-economic development of the three EU regions and Europe as a whole. However, more in-depth analysis on the economic impact is needed, e.g. number of patents coming directly as a result of innovative research from the three centers, utility models, the topics and the businesses affected, and a related network analysis with partners across Europe and all over the world. The lack of such analysis is a weakness and limitation of this study but also an opportunity for further investigations in this direction. In addition to he direct economic effects of such patents, utility models, and business innovations, the global network analysis could identify also the indirect benefits to the academic and business communities as well as to the partner institutions, as these connections are mutually beneficial. Another future avenue of research would be in-depth studies on other aspects of the centers' impact on society, such as networking with institutional entities, creating operational guidelines, policy briefs, decision-support tools, as well as linking with other stakeholders by organizing workshops, living labs, training activities, and other outreach measures.

An important factor for the development of the local ecosystems, catalyzed by the three centers, is development is the support from local and central Governments of Germany, Flanders, and Bulgaria. The Governments of these countries have realized the need of investing in research and innovation to create such local hubs, which are drivers of scientific, economic, and social development not only for the local regions but for the countries and Europe as a whole. These are really good practices and examples that other countries, especially the new EU member states, can learn from.

In conclusion, this case study is an example how scientists who return from North to South-West can establish a new center of excellence which can stimulate knowledge circulation, partnership between regions, and catalyze the socio-economic development of South-East and Europe as a whole.

## Data Availability

The original contributions presented in the study are included in the article/Supplementary material, further inquiries can be directed to the corresponding author.
