# Supplementary material for: Impact of leading plant research centers of excellence on the scientific and socio-economic development in Europe
Source: Front Res Metr Anal. 2026 Apr 16;11:1770226. doi: 10.3389/frma.2026.1770226 (PMC13128557; doi:10.3389/frma.2026.1770226)
Supplement: Supplementary file 1 [file Data_Sheet_1.zip › Supplementary Figure 1.pdf]

A

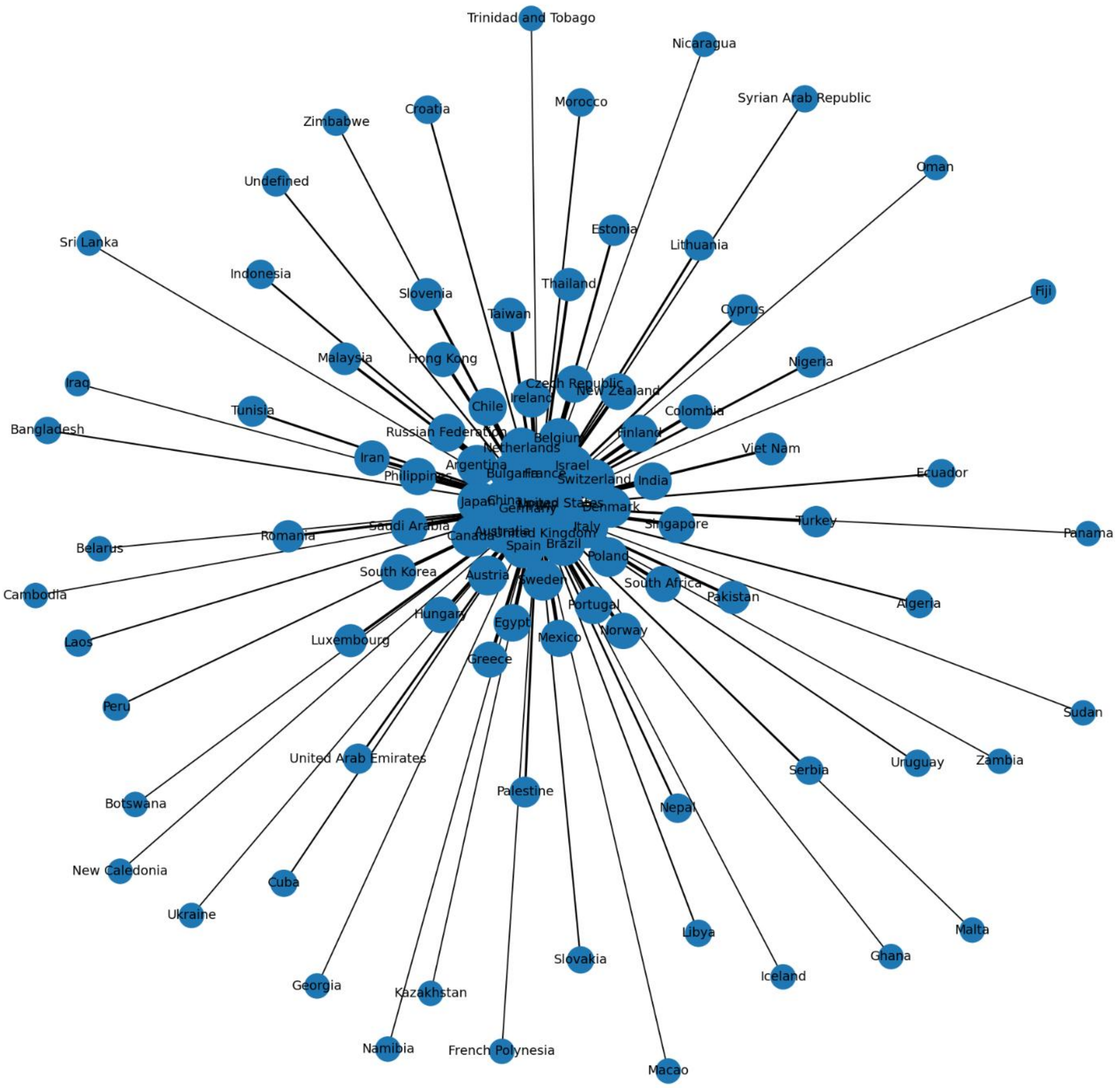

Supplementary Figure 1. Network analysis of the publication partner countries of the three research centers. A, partners of MPIMP; B, partners of PSB; C, partners of CPSBB. The three centers are positioned in the middle of the three networks. The countries with most partner organizations are closer to the centers, whereas the countries with least number of partner organizations are positioned farthest.

B

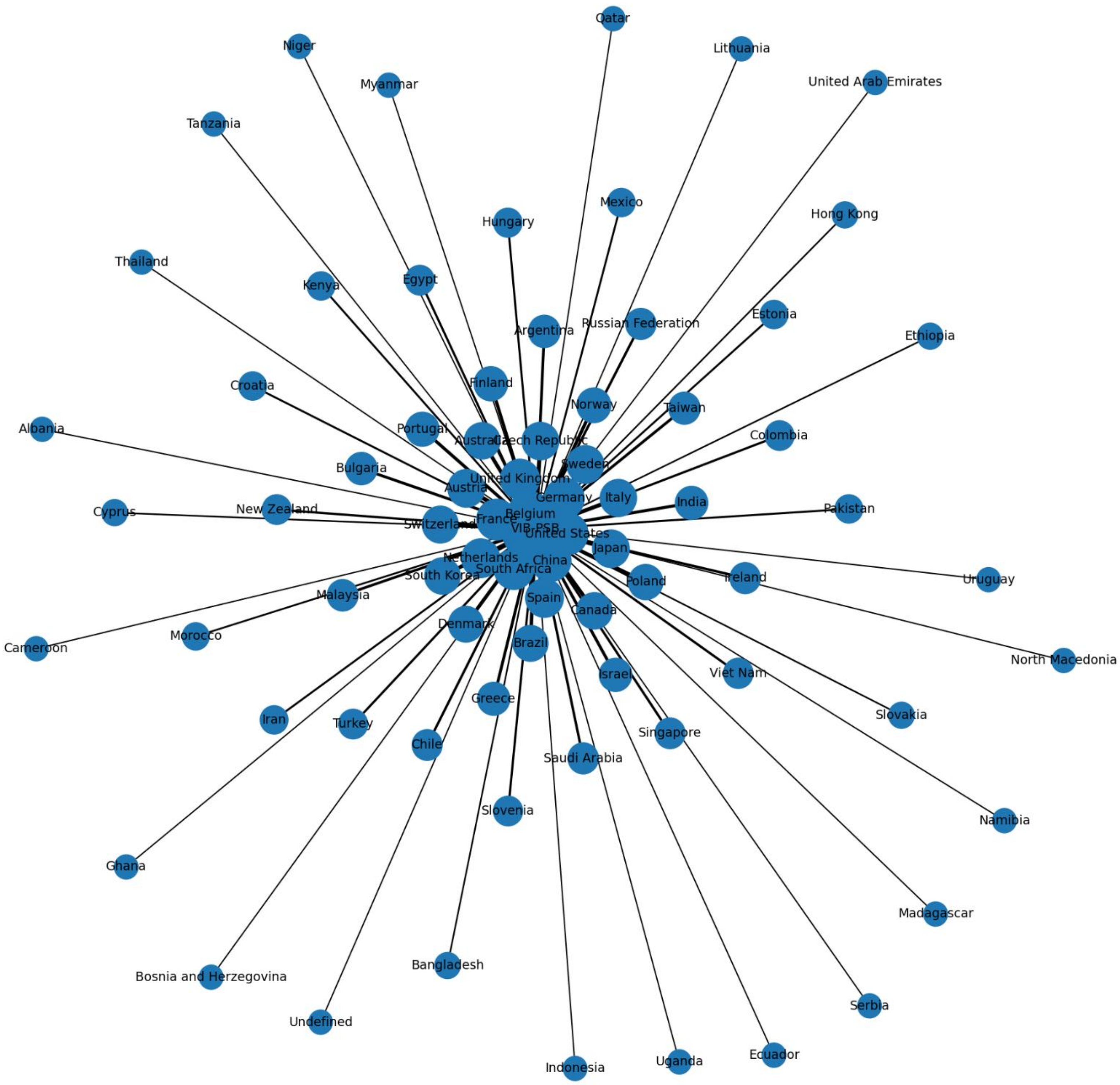

Supplementary Figure 1. Network analysis of the publication partner countries of the three research centers. A, partners of MPIMP; B, partners of PSB; C, partners of CPSBB. The three centers are positioned in the middle of the three networks. The countries with most partner organizations are closer to the centers, whereas the countries with least number of partner organizations are positioned farthest.

C

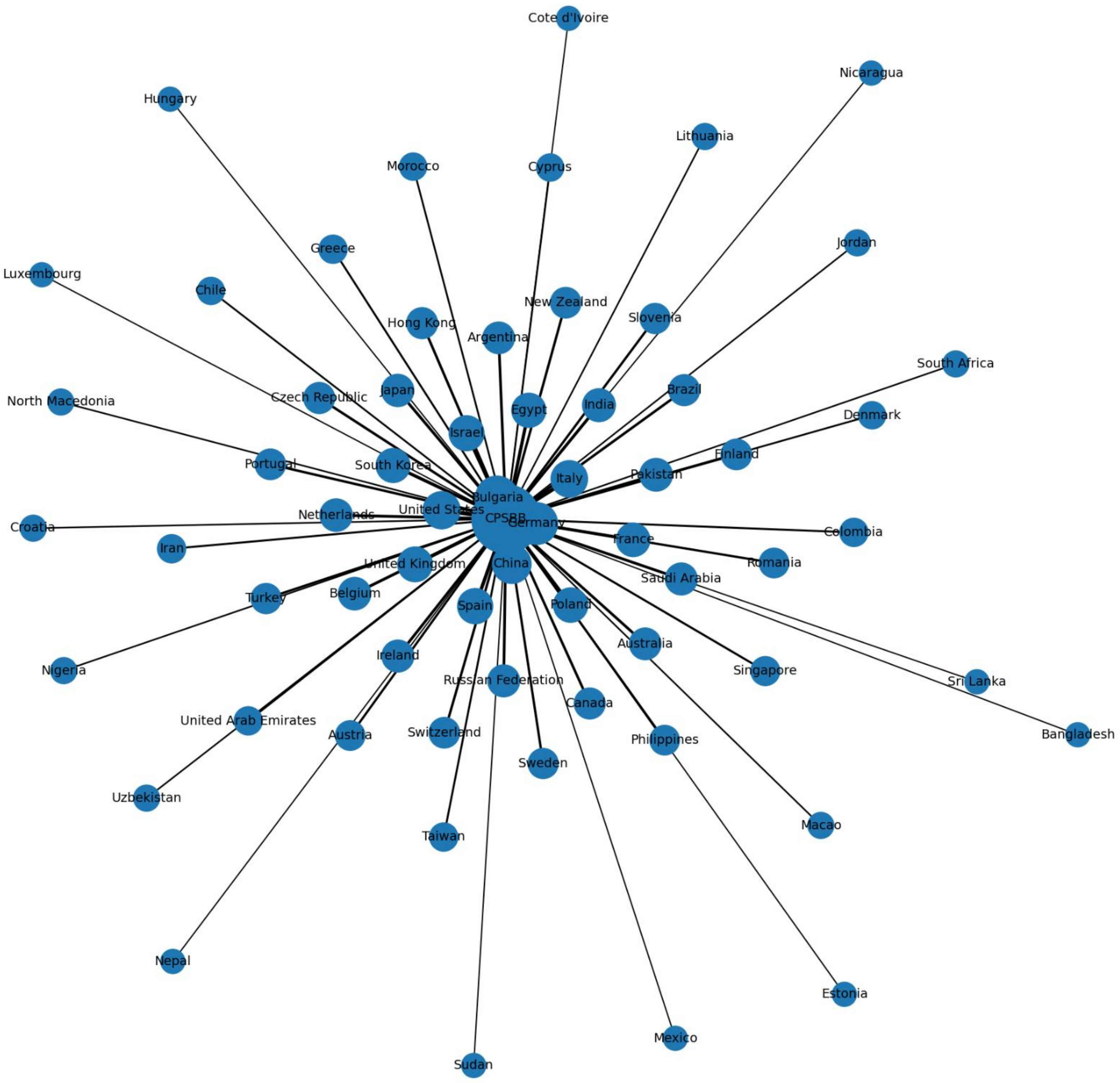

Supplementary Figure 1. Network analysis of the publication partner countries of the three research centers. A, partners of MPIMP; B, partners of PSB; C, partners of CPSBB. The three centers are positioned in the middle of the three networks. The countries with most partner organizations are closer to the centers, whereas the countries with least number of partner organizations are positioned farthest.
